# Severe Colonic Dysmotility and Constipation Leading to Hydronephrosis in Advanced Parkinson’s Disease: A Case Report and Literature Review

**DOI:** 10.7759/cureus.77035

**Published:** 2025-01-06

**Authors:** Giovannie Isaac-Coss, Swathi Kavuri, Jayalekshmi Jayakumar, Zoe Post, Atsushi Sakuraba

**Affiliations:** 1 Gastroenterology and Hepatology, Rush University Medical Center, Chicago, USA; 2 Internal Medicine, The Brooklyn Hospital Center, New York, USA

**Keywords:** alpha-synuclein, autonomic dysfunction, chronic constipation, colonic dysmotility, enteric nervous system, gastrointestinal motility disorders, hydronephrosis, parkinson's disease, severe constipation, urinary tract compression

## Abstract

Parkinson's disease (PD) is a progressive neurodegenerative disorder characterized by motor symptoms and significant non-motor complications, including gastrointestinal disturbances. Constipation, which affects up to 80% of PD patients, is one of the most common and debilitating gastrointestinal symptoms. This case report describes a 77-year-old female with advanced PD, who presented multiple times to the emergency department with recurring symptoms of vomiting, abdominal pain, and severe abdominal distension. Imaging studies revealed rectosigmoid colonic dilation, fecal impaction, and right hydronephrosis and hydroureter, caused by external compression from the distended colon - a rare complication in PD. The condition was clinically diagnosed as colonic dysmotility, linked to alpha-synuclein deposition in the enteric nervous system. Management included a unique combination of rectal tube placement, manual disimpaction, and pharmacological therapy, which successfully alleviated her symptoms and resolved the associated complications. Despite the effective management of symptoms, no current therapies exist to prevent or address the underlying alpha-synuclein-related colonic dysmotility. This case underscores the need for early recognition of severe colonic dysmotility in PD and highlights the importance of further research into targeted treatments that address the complex pathophysiology of gastrointestinal dysfunction in PD.

## Introduction

Parkinson’s disease (PD) is a progressive neurodegenerative disorder primarily characterized by motor symptoms such as tremors, bradykinesia, rigidity, and postural instability. However, non-motor symptoms also play a significant role in disease progression and quality of life. Among these, gastrointestinal (GI) disturbances, especially constipation and colonic dysmotility, are prevalent and can severely affect patients. Up to 80% of individuals with PD experience constipation, which may significantly contribute to discomfort and disability [1-3]. These GI symptoms, often overlooked or underreported, are increasingly recognized as a crucial aspect of PD management.

Colonic dysmotility, a common manifestation of gastrointestinal dysfunction in PD, is primarily caused by alpha-synuclein deposition in the enteric nervous system (ENS), which impairs normal motility. The pathological accumulation of alpha-synuclein in the myenteric plexus and submucosal ganglia of the GI tract disrupts peristalsis, leading to delayed gastric emptying and constipation. This dysfunction is exacerbated by dopaminergic treatments, which, while essential for managing motor symptoms, often worsen constipation. As a result, patients with PD frequently experience a progressive decline in bowel function, leading to complications such as fecal impaction, colonic distension, and, in severe cases, pseudo-obstruction [4-8].

Hydronephrosis is a rare but serious complication of untreated or underrecognized colonic dysmotility in PD. When colonic distension and fecal impaction occur, the large bowel can exert external pressure on the urinary tract, leading to obstructive uropathy and renal impairment. Hydronephrosis, the swelling of a kidney due to the build-up of urine, can result from this external compression. If left unrecognized or untreated, hydronephrosis can lead to severe complications such as kidney damage, infection, and sepsis. Prompt diagnosis and management of this complication are crucial to preventing permanent renal damage and further compromising the patient’s quality of life.

While the prevalence of constipation in PD is well documented, there is a lack of comprehensive research on colonic dysmotility and its associated complications [9]. The limited literature surrounding the management of severe constipation in PD highlights the need for better diagnostic and therapeutic approaches. This case report focuses on a patient with advanced PD who developed hydronephrosis as a direct result of untreated colonic dysmotility. The patient’s case underscores the importance of early identification and intervention to mitigate these complications, which are often overlooked in clinical practice.

This case also draws attention to the broader issue of colonic dysmotility in PD, a condition that is frequently underappreciated in both clinical and research settings. The implications of these GI disturbances are profound, as they significantly impact patient care, treatment adherence, and overall disease management. By examining the intersection of severe constipation, colonic distension, and hydronephrosis, this case highlights the need for more targeted research and clinical awareness to improve outcomes for PD patients affected by GI dysfunction.

## Case presentation

A 77-year-old female with a do not resuscitate/do not intubate (DNR/DNI) order and significant medical history, including hypertension, PD, sacral vertebral fractures (status post anterior-posterior decompression and fusion), wheelchair-bound (after spinal surgery), and sacral ulcers, presented to the emergency department with a one-day history of vomiting and generalized abdominal pain. The emesis was primarily food, non-bloody, and non-bilious. Her last bowel movement, described as liquid, occurred two days prior according to her home health attendant (HHA). The patient is passing flatus. She has a history of multiple admissions for similar complaints and is wheelchair-bound and unable to walk. In January 2024, she was similarly admitted and advised to continue a bowel regimen of Senna, MiraLAX, and Colace, although adherence at home is uncertain. A review of systems was positive for vomiting, generalized abdominal pain, and constipation and negative for chills, headache, chest pain, dyspnea, diarrhea, and dysuria. Her home medications include enalapril, amlodipine, carbidopa-levodopa 25/250 mg every six hours, and lactulose 15 mL of 10 g/15 mL every six hours as needed. Her last colonoscopy, performed over five years ago, was normal, and the timing for the next colonoscopy is uncertain.

The patient lives at home with her husband and has been wheelchair-bound since 2020 after her spinal surgery. She requires 24-hour care from an HHA. Due to her physical limitations from PD, including impaired mobility and difficulty with daily activities, the patient faces significant challenges in maintaining her bowel regimen. Her ability to adhere to prescribed treatments, including medications and dietary adjustments, is likely impacted by her reliance on caregivers for assistance with daily tasks. The patient's physical and functional impairments, combined with caregiver limitations, may contribute to the recurrence of her gastrointestinal symptoms, particularly constipation and fecal impaction.

The patient denied smoking, alcohol use, or illicit substance use. Family history was non-contributory.

Upon presentation, her vital signs were temperature 98.3°F, heart rate 114 beats per minute, blood pressure 153/114 mmHg, oxygen saturation 98% on room air, and respiratory rate 20 breaths per minute. Physical examination revealed an alert and oriented patient, lying comfortably in bed. Examination of her extremities showed rigid tone and mild tremors bilaterally. The abdominal exam noted a soft, non-tender, distended abdomen with normoactive bowel sounds and no guarding or rebound tenderness. A large, reducible 8 cm x 8 cm hernia was observed in the left lower abdomen.

The diagnosis of PD in this patient was made based on clinical features, as reported by her family members (daughter and husband). They recall that the patient was diagnosed with PD in her 40s. Unfortunately, they were unable to provide further details regarding the specific diagnostic methods used at that time, such as whether a dopamine transporter (DaT) scan or skin biopsy for alpha-synucleinopathy was performed, as these events occurred many years ago. Her Unified Parkinson's Disease Rating Scale (UPDRS) score was 173/199 (86.93%), based on historical data, physical examination, and primary care physician (PCP) notes. A review of the patient's current medical records, specifically from her PCP, confirms that she is currently being prescribed carbidopa-levodopa (25/250 mg, four times daily). The patient has not seen a neurologist in recent years and prefers to continue with her current medication regimen without pursuing additional follow-up care or diagnostic evaluations.

**Figure 1:**
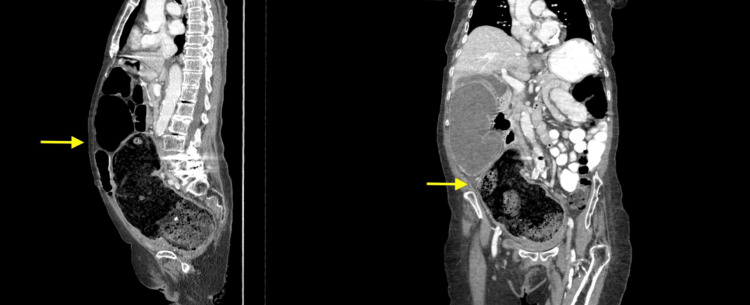
CT abdomen pelvis without contrast was read as marked colonic distension, particularly pronounced in the rectosigmoid region, which is consistent with constipation and fecal impaction (yellow arrow).

**Figure 2:**
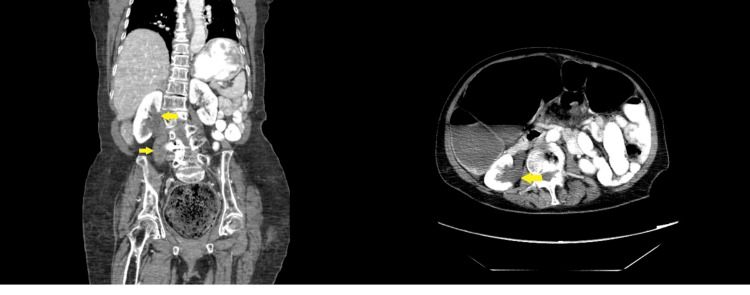
CT abdomen pelvis showing right hydronephrosis and hydroureter, likely resulting from external compression due to the significant colonic distension.

In the Emergency Department (ED), the patient was administered Zofran. CT imaging of the abdomen and pelvis demonstrated severe distension of the colon, particularly in the rectosigmoid area, consistent with constipation and fecal impaction (yellow arrow). In addition, there was evidence of right hydronephrosis and hydroureter, likely due to external compression from the distended colon. This condition occurs when the enlarged colon presses against the ureter, causing a backup of urine in the kidney (Figures 1-2).

Laboratory results revealed hyperkalemia (5.5 mmol/L), leukocytosis (11.1 K/μL), and elevated aspartate aminotransferase (37 U/L). Lactic acid was normal (1.6 mmol/L). Urinalysis showed moderate urine leukocytes and blood, with microscopy revealing numerous red blood cells and 20 to 50 white blood cells per high-power field (HPF). Urine culture grew 25,000 colonies/mL of beta-hemolytic streptococci group B. Blood cultures did not grow any organisms throughout the admission (Table 1).

**Table 1:** Relevant laboratory workup H = high

Relevant labs	Results	Reference
Complete blood count	White blood cell count = 11.1 K/cmm (H)	(4.8–10.8 K/cmm)
	Hemoglobin = 16.5 g/dL (H)	(13.1–15.5 g/dL)
	Hematocrit = 49 % (H)	(39%–47%)
	Platelets = 374 K/cmm	(130–400 K/cmm)
Basic metabolic panel	Sodium = 136 MMOL/L	(136–145 MMOL/L)
	Potassium = 5.5 MMOL/L	(3.5–5.1 MMOL/L)
	Creatinine = 0.7 MG/DL	(0.7–1.3 MG/DL)
	Magnesium = 2.2 MG/DL	(1.6–2.6 MG/DL)
	Phosphorus = 3.4 MG/DL	(2.3–4.7 MG/DL
Hepatic function panel	Bilirubin total = 0.8 MG/DL	(0.2–1.2 MG/DL)
	Bilirubin direct = 0.2 MG/DL	(0.0–0.5 MG/DL)
	Aspartate aminotransferase = 37 U/L (H)	(8–34 U/L)
	Alanine transaminase = 10 U/L	(6–55 U/L)
	Albumin = 4.4 G/DL	(3.5–5.0 G/DL)
	Alkaline phosphatase = 106 U/L	(40–150 U/L)
	Total protein serum = 9.3 G/DL (H)	(6.4–8.3 G/DL)
Microbiology	Blood cultures = No growth	No growth

**Figure 3:**
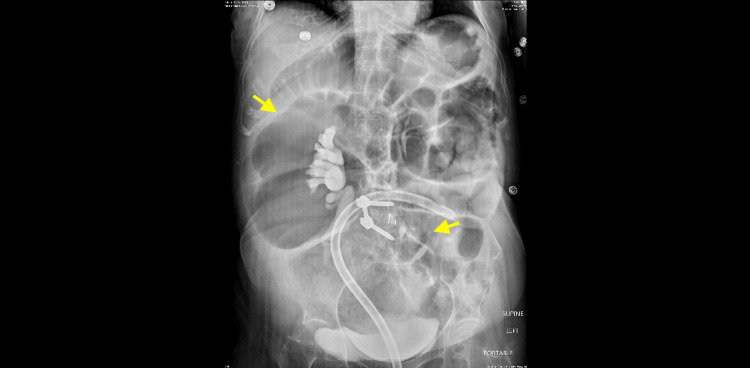
X-ray of the abdomen showing massive amount of stool in the sigmoid colon and rectum, reflecting impaction (yellow arrows). The rectal tube tip projects over the left iliac crest.

Surgery was consulted and recommended manual disimpaction, pharmacologic stool regimen, rectal tube placement, and serial abdominal examinations. Abdominal X-ray confirmed a large, stable fecal mass in the rectum, with the rectal tube in place (Figure 3).

The patient was admitted to the hospital for further management. Upon rectal tube insertion, two pounds of stool were successfully removed. Afterward, the rectal tube was removed. Urology was consulted due to new onset right hydroureter and hydronephrosis and recommended a follow-up ultrasound 72 hours after addressing the fecal impaction and constipation. Gastroenterology was also consulted and recommended tap water enemas along with pharmacologic stool regimen.

A follow-up ultrasound of the kidneys showed the right kidney measuring 10.2 x 3.4 x 5 cm and the left kidney measuring 8.9 x 3.8 x 3.2 cm, with normal renal parenchyma and no evidence of renal mass or hydronephrosis. A non-obstructive renal calculus, up to 1 cm in size, was noted in the right mid-pole. The urinary bladder was partially distended with no masses observed.

Aggressive bowel management led to a significant reduction in gas and fecal impaction, resulting in the resolution of hydronephrosis and abdominal pain. The patient was advised to follow up with Urology as an outpatient for the non-urgent, non-obstructing 1 cm right kidney stone. A digital rectal examination revealed a normal rectal sphincter tone, a dilated rectum, and no masses. The gastroenterology team cleared the patient for discharge, with instructions for primary care and gastroenterology follow-up, daily enemas as needed, Senna daily, and Colace twice per day. Medication compliance was emphasized to prevent future hospitalizations. The patient declined follow-up with Neurology, preferring to continue with her current medication regimen without further neurologic evaluation.

## Discussion

Pathophysiology

PD is a progressive neurodegenerative disorder that primarily impacts motor control, but also leads to a range of non-motor symptoms, including GI disturbances. Among the GI issues, constipation and colonic dysmotility are among the most common and debilitating symptoms. These symptoms are often linked to both the direct pathological effects of PD on the enteric nervous system (ENS) and the autonomic dysfunction commonly observed in PD patients. A key contributor to these issues is the deposition of alpha-synuclein in the ENS, which interferes with gastrointestinal motility. This abnormal accumulation of alpha-synuclein is well-documented in PD, with studies showing that these deposits disrupt the functioning of the gastrointestinal system, contributing to severe constipation and colonic dysmotility [6,7,8].

In our case, the patient’s significant constipation and colonic distension are consistent with these pathophysiological mechanisms. The deposition of alpha-synuclein in the enteric neurons of the gut likely impaired peristalsis, resulting in severe colonic dysmotility [7]. In addition, the use of dopaminergic medications, which are essential for managing PD’s motor symptoms, can exacerbate constipation by reducing gut motility. As such, the therapeutic management of PD-related constipation must strike a balance between alleviating motor symptoms and mitigating the adverse effects on bowel function [6,9].

Diagnostic challenges

The diagnosis of colonic dysmotility in PD is typically based on clinical evaluation, imaging, and sometimes specialized diagnostic tests. For our patient, diagnostic imaging (abdominal X-ray) revealed colonic distension and impaction, which confirmed the diagnosis of severe constipation. While specialized tests such as colonic manometry, scintigraphy, and barium enema are valuable tools for assessing colonic motility, they were not pursued in this case due to the patient’s palliative care approach. Given the advanced stage of the patient’s disease and the focus on symptom management rather than exhaustive diagnostic workup, only basic imaging was used to guide treatment decisions. This is consistent with other studies, which have emphasized that many PD patients are managed with symptom-directed approaches, particularly in the advanced stages of the disease [5,9].

Management strategies

Effective management of severe colonic dysmotility in PD requires a combination of pharmacological and mechanical interventions. In this case, a multifaceted approach was employed, including the use of pharmacologic agents (Senna, MiraLAX, and lactulose), manual disimpaction, and rectal tube placement. This combination was crucial in addressing the patient’s severe fecal impaction and colonic dilation. The rectal tube, in particular, was essential for relieving the external compression caused by the distended colon, which was contributing to the patient’s hydronephrosis [5].

Pharmacologic treatment for PD-related constipation typically includes laxatives and stool softeners. However, care must be taken when prescribing these medications due to their potential interactions with dopaminergic agents. For example, prokinetic agents, such as metoclopramide or prucalopride, can help promote colonic motility but may exacerbate motor symptoms of PD if used alongside dopaminergic medications [9]. As such, any medication regimen must be carefully tailored to the patient’s overall clinical picture, considering both the GI and motor manifestations of PD.

In addition, non-pharmacologic interventions, such as dietary modifications, adequate hydration, and regular physical activity, are recommended as part of a holistic management approach to prevent constipation in PD patients [10]. However, in patients with advanced PD and significant physical limitations, as seen in this case, these lifestyle interventions can be challenging to implement and may require the support of caregivers.

Complications

One of the unique aspects of this case was the development of right-sided hydronephrosis, likely caused by external compression from colonic distension. Hydronephrosis, a condition in which the kidney becomes swollen due to urine buildup, is a rare complication of severe colonic dysmotility. In this patient, the marked colonic dilation led to compression of the urinary tract, resulting in hydronephrosis and hydroureter. This highlights the serious consequences of untreated severe constipation in PD patients. Prompt intervention, including manual disimpaction and rectal tube placement, resolved the hydronephrosis and alleviated the patient’s symptoms, underscoring the importance of timely management to prevent such complications.

Other potential complications of colonic dysmotility in PD include bowel obstruction, perforation, rectal prolapse, and even sepsis. The resolution of hydronephrosis in this case illustrates the efficacy of an aggressive treatment approach and the importance of preventing the progression of GI complications [6,9,11-13].

Preventive measures

Preventing colonic dysmotility in PD involves regular monitoring of bowel function, dietary modifications, and promoting physical activity. Studies have shown that high-fiber diets and adequate fluid intake are essential in managing constipation in PD patients [10]. Moreover, for patients at high risk of severe constipation, such as those with advanced PD, proactive adjustments to bowel regimens and early initiation of pharmacologic interventions are critical to preventing the recurrence of symptoms and complications [9].

In this case, the patient’s functional limitations - such as her need for 24-hour HHA care and her advanced disease stage - significantly influenced both her treatment adherence and overall care. The patient's mobility challenges and reliance on caregivers made it difficult to maintain an optimal bowel regimen, which likely contributed to the recurrence of severe constipation. This reinforces the need for a patient-centered approach, with caregivers playing a pivotal role in ensuring adherence to the treatment plan [5,6].

Future directions

Future research should continue to explore the mechanisms behind colonic dysmotility in PD, particularly in relation to alpha-synuclein deposition and autonomic dysfunction. Studies examining the long-term outcomes of various treatment strategies, including the use of prokinetic agents and mechanical interventions, could help refine management approaches for PD patients with GI symptoms. Additionally, further investigation into the interaction between dopaminergic medications and GI motility may help optimize the balance between managing motor and non-motor symptoms in PD [8,9].

This case also highlights the importance of early recognition and intervention in preventing severe complications of colonic dysmotility in PD. By integrating pharmacological and mechanical treatments into a comprehensive management plan, healthcare providers can improve patient outcomes and reduce hospitalizations. A more personalized approach that considers the patient’s functional status and caregiver support is crucial for effective long-term management.

## Conclusions

This case report highlights the challenging management of severe colonic dysmotility complicated by hydronephrosis in a patient with advanced PD. The external compression of the urinary tract due to colonic distension underscores the profound impact gastrointestinal dysfunction can have on multiple organ systems. The management approach, which included rectal tube placement, manual disimpaction, enemas, and pharmacologic therapy, successfully alleviated symptoms and resolved the complication. However, there remains no current treatment to prevent or reverse the deposition of alpha-synuclein in the gastrointestinal tract, a key factor in colonic dysmotility in PD. This case underscores the need for further research into targeted therapies that could mitigate alpha-synuclein-related gastrointestinal dysfunction. Such advancements would have the potential to significantly enhance patient outcomes and quality of life for individuals affected by these debilitating complications of PD.

## References

[1] Jankovic J (2008). Parkinson’s disease: clinical features and diagnosis. J Neurol Neurosurg Psychiatry.

[2] Chaudhuri K, Schapira A (2009). Non-motor symptoms of Parkinson’s disease: dopaminergic pathophysiology and treatment. Lancet Neurol.

[3] Fasano A, Visanji NP, Liu LW (2015). Gastrointestinal dysfunction in Parkinson’s disease. Lancet Neurol.

[4] Natale G, Pasquali L, Ruggieri S, Paparelli A, Fornai F (2008). Parkinson's disease and the gut: a well known clinical association in need of an effective cure and explanation. Neurogastroenterol Motil.

[5] Pfeiffer R (2011). Gastrointestinal dysfunction in Parkinson’s disease. Parkinsonism Relat Disord.

[6] Hamilton JP, Chen MC, Gotlib IH (2013). Neural systems approaches to understanding major depressive disorder: an intrinsic functional organization perspective. Neurobiol Dis.

[7] Braak H, Devos RA, Bohl J (2006). Gastric alpha-synuclein immunoreactive inclusions in Meissner’s and Auerbach’s plexuses in cases staged for Parkinson’s disease-related pathology. Neurosci Lett.

[8] Shannon KM, Keshavarzian A, Mutlu E (2012). Alpha-synuclein in colonic submucosa in early untreated Parkinson’s disease. Mov Disord.

[9] Gjerde CI, Muller B, Skeie GO (2015). Prevalence of constipation and its association with Parkinson disease: a 3-year follow-up study. Neurogastroenterol Motil.

[10] Sung HY, Park JW, Kim JS (2014). The frequency and severity of gastrointestinal symptoms in patients with early Parkinson's disease. J Mov Disord.

[11] Edwards LL, Quigley EM, Pfeiffer RF (1994). Gastrointestinal symptoms in Parkinson disease: 18-month follow-up study. Mov Disord.

[12] Ueki A, Otsuka M (2004). Life style risks of Parkinson’s disease: association between decreased water intake and constipation. J Neurol.

[13] Abbott RD, Petrovitch H, White LR (2001). Frequency of bowel movements and the future risk of Parkinson’s disease. Neurology.

